# rs762855 single nucleotide polymorphism modulates the risk for diffuse-type gastric cancer in females: a genome-wide association study in the Korean population

**DOI:** 10.1007/s10120-024-01575-6

**Published:** 2025-01-25

**Authors:** Kyungtaek Park, Cheol Min Shin, Nayoung Kim, Sungho Won, Chin-Hee Song, Jung Hun Ohn, Sejoon Lee, Ji Hyun Park, Ga-Eun Yie, Seung Joo Kang, Joo Sung Kim, Dong Ho Lee

**Affiliations:** 1 Institute of Health and Environment, Seoul National University, Seoul, Republic of Korea; 2 Department of Internal Medicine, Seoul National University Bundang Hospital, Seongnam, Gyeonggi-Do South Korea; 3 Research Center for Sex- and Gender-Specific Medicine, Seoul National University Bundang Hospital, 173-82, Gumi-ro, Bundang-gu, Seongnam, Gyeonggi-Do South Korea; 4 Department of Internal Medicine and Liver Research Institute, Seoul National University College of Medicine, Seoul, South Korea; 5 Department of Public Health Sciences, Seoul National University, Seoul, South Korea; 6 Interdisciplinary Program of Bioinformatics, Seoul National University, Seoul, Korea; 7 Precision Medicine Center, Seoul National University Bundang Hospital, Seongnam, South Korea; 8 Department of Internal Medicine and Healthcare Research Institute, Healthcare System Gangnam Center, Seoul National University Hospital, Seoul, Korea

**Keywords:** Gastric cancer, Genome-wide association study, Lauren classification, Sex differences

## Abstract

**Background:**

Intestinal-type gastric cancer (IGC) and diffuse-type gastric cancer (DGC) exhibit different prevalence rates between sexes. While environmental factors like *Helicobacter pylori* infection and alcohol consumption contribute to these differences, they do not fully account for them, suggesting a role for host genetic factors.

**Methods:**

We conducted a meta-analysis to explore associations between single nucleotide polymorphisms (SNPs) and the risk of IGC or DGC. The analysis included the SNUBH cohort (998 participants: 159 DGCs, 303 IGCs, 4,962,361 variants) and the GC_HC cohort (6,233 participants: 389 DGCs, 405 IGCs, 4,541,617 variants). Significant variants were validated in the SNUBH2_AA cohort (5,511 participants: 40 DGCs, 49 IGCs, 3,668,632 variants).

**Results:**

The meta-analysis identified that rs762855 (chr4:3,074,795; hg19) is significantly associated with DGC risk in females (OR [95% CI]: 1.758 [1.438–2.150], *P* = 3.91 × 10^–8^), a finding replicated in the SNUBH2_AA datasets (OR [95% CI]: 3.356 [1.031–10.92], *P* = 4.43 × 10^–2^). Gene-set and transcriptomic analyses revealed that the Myb/SANT DNA Binding Domain Containing 1 (*MSANTD1*) gene is significantly linked to DGC susceptibility in females. In addition, Mendelian randomization analyses suggested that increased serum total protein and non-albumin protein (NAP) levels elevate DGC risk in females (*P* < 0.05), but not in males.

**Conclusion:**

The rs762855 SNP, *MSANTD1,* and serum NAP levels are associated with DGC risk in Korean females.

**Supplementary Information:**

The online version contains supplementary material available at 10.1007/s10120-024-01575-6.

## Introduction

Gastric cancer (GC) was the fifth most frequently diagnosed cancer, with the fifth highest cancer-related mortality worldwide in 2022 [1]. Besides *Helicobacter pylori* (*H. pylori*), smoking, alcohol consumption, preserved food consumption, low intake of fresh vegetables or fruits, and genetic susceptibility may independently or jointly contribute to GC risk [2].

Lauren’s classification of GC histologically divides it into two major subtypes, which have clear differences in epidemiology and pathogenesis [3]. Intestinal-type GC (IGC) is classically associated with chronic *H. pylori* infection and a terminal event in the neoplastic progression termed Correa’s cascade in the following sequence: chronic gastritis, atrophic gastritis (AG), intestinal metaplasia (IM), and dysplasia [4–6]. IGC typically originates in the distal stomach, with most IGC patients being males and older adults [7]. Conversely, diffuse-type GC (DGC) occurs at a younger age, predominantly in females, and commonly originates in the proximal stomach [7]. DGC may have a stronger genetic contribution than IGC [7]. Recently, the prevalence of *H. pylori* has declined due to improved hygiene and active eradication treatment. Thus, the incidence of IGC has reduced over the last half-century worldwide. However, the incidence of DGC remains stable and is even increasing in certain areas [8]. Moreover, DGC has a significantly poorer prognosis than IGC, partly because of more advanced stage at time of diagnosis [9].

Sex differences in the risk of GC are known to exist. Female sex hormones might protect against GC [10]. IGC predominates males, attributable to higher exposure to environmental risk factors, including smoking, alcohol consumption, and dietary carcinogens in males [7]. In contrast, DGC can occur in *H. pylori*-positive patients without atrophy or IM and is more common among young females [11].

Genome-wide association studies (GWAS) have reported significant associations of GC with single nucleotide polymorphisms (SNPs) of rs2294008 in the *Prostate Stem Cell Antigen* (*PSCA*) gene and rs4072037 in the *Mucin 1, Cell Surface Associated* (*MUC1*) gene, with findings replicated across various populations, including East Asians and Caucasians [12]. SNPs within the *Cut Like Homeobox* *2* (*CUX2*) genes region on chromosome 12q24.11–12 were found to be significantly associated with GC in a large Japanese cohort [13]. Recently, we also reported that rs2671655 increased the risk of GC in the presence of *H. pylori* infection, especially in patients with DGC, by regulating the expression of several genes, such as *Zinc Finger Protein 652* (*ZNF652*) and *Speckle Type BTB/POZ Protein* (*SPOP*) which consequently modify susceptibility to GC [12].

To date, few studies have comprehensively assessed the effects of not only *H. pylori*, but also Lauren classification, genetic susceptibility, and sex differences on GC. Against this background, we attempted to identify genetic variations associated with GC risk according to sex and histological type by combining and analyzing additional cohorts with our previous study.

## Methods

### GWAS using SNUBH and GC_HC cohorts

The Seoul National University Bundang Hospital (SNUBH) cohort comprises 1,216 participants, consisting of 610 patients with GC and 606 non-cancer controls, with ages ranging from 26 to 80 years, who were recruited from the enrolled participants who visited the Digestive Disease Center, SNUBH. In our previous study [12], we conducted quality control (QC) procedures, guided by suggestions from Anderson et al. (Supplementary Fig. S1) [14], with using plink (v1.90b3.44)[15]. Following QC and imputation, we retained 4,962,361 variants from 968 participants (527 GC patients and 441 non-cancer controls) with complete data for cancer status, sex, age, *H. pylori* status, and smoking status.

Furthermore, from the National Biobank of Korea (NBK) (https://biobank.nih.go.kr/cmm/main/engMainPage.do), we obtained genetic data from 803 patients with GC, including 394 with DGC and 409 with IGC, whose genotypes were sequenced using the Affy 6.0 platform. In addition, within the Korean Genome and Epidemiology Study (KoGES) database, housed in the NBK, the control group comprised 3,693 urban residents from KoGES_health examinee (KoGES_HEXA) and 1,816 rural residents from KoGES_cardiovascular disease association study (KoGES_CAVAS). These databases were utilized to investigate susceptibility loci for chronic and cardiovascular diseases, respectively, with genotyping conducted using the same platform. We referred to the combined database of these three cohorts as GC_HC throughout the text. In GC_HC, we compared the genotypes of 803 patients diagnosed with DGC or IGC to those of the 5,509 participants. QC procedures were conducted separately for each dataset, following the same procedure as the SNUBH cohort, except for variant selection, which included only variants with a minor allele frequency (MAF) > 0.05 (Supplementary Fig. S1). The datasets were then merged. As the sequenced readings were aligned to the hg18 human reference genome, we lifted them over to the hg19 human reference genome using the LiftOver tool [16]. We performed QC procedures on the merged data, using the same cutoff values as in the previous steps. We generated a multi-dimensional scaling (MDS) plot to evaluate genetic heterogeneity among the three datasets, with variants pruned using the ‘*–indep-pairwise 50 5 0.2’* option in plink (Supplementary Fig. S2A).

Next, we imputed the variants using the Michigan Imputation Server (v1.1) [17], employing the same parameters as those used for the SNUBH cohort (Supplementary Fig. S1). We removed variants with an imputation quality score < 0.8 and a MAF < 0.05 following imputation. Ultimately, we retained 4,541,617 variants from 6,233 participants (794 patients with GC, including 389 with DGCs and 405 with IGCs, and 5,439 controls.

We sub-grouped the participants based on the Lauren classification (diffuse or intestinal) and sex (female or male), and compared the variants of GC patients with those of controls within each cluster. For instance, in the SNUBH cohort, we compared the variants of 72 female patients with DGC to those of 248 female non-cancer controls, whereas in the GC_HC cohort, we compared the variants of 165 female patients with DGC to those of 2,958 female controls (Supplementary Fig. S1). Furthermore, female participants were stratified into two groups according to age (≥ 50 y or < 50 y), and variants were compared between DGC patients and controls in each group. The number of female patients with DGC and controls aged ≥ 50 y were 40 and 161 in the SNUBH cohort, and 82 and 1,953 in the GC_HC cohort. The number of female patients with DGC and controls aged < 50 y was 32 and 87 in the SNUBH cohort and 83 and 1,005 in the GC_HC cohort.

GWAS was conducted using SAIGE (v0.44.6.4) [18] in R (v4.1.1) environment with its default options, except for increasing the value of ‘*maxiterPCG*’ option from 500 to 1,000, and adjusting for the effects of sex (only when both sexes of participants were included), age, *H. pylori* status, smoking status, and the top four principal component (PC) scores in the SNUBH cohort, and sex, age and the top four PC scores in the GC_HC cohort. Summary statistics were utilized for meta-analysis with inverse variance weighting using METAL (version released on 2011–03-25) [19]. The qqman (v0.1.8) R package was used to generate Manhattan and quantile–quantile (QQ) plots [20]. The LocusZoom plot was created at http://locuszoom.org/ with default settings, except for designating the LD population as 1000G Nov 2014 ASN [21].

### Validation of significant variants using the SNUBH2 and KoGES_Ansan/Ansung cohorts

We sequenced additional 102 patients with GC using the Korea Biobank Array to validate the results of the meta-analysis; the patients were selected from the same patient pool as the SNUBH cohort (referred to as SNUBH2). Within KoGES, genotypes from 5,493 participants in the KoGES_Ansan/Ansung cohort were sequenced utilizing the Korea Biobank array and served as controls in our study who were recruited to examine the associations between chronic diseases and lifestyle or diet from two cities in Korea. The combined cohorts were referred as SNUBH2_AA throughout the text. We employed the same QC and imputation steps as in the GC_HC cohort, resulting in the retention of 3,668,632 variants in 5,511 participants (101 patients with GC and 5,410 controls) (Supplementary Fig. S1). Among these participants, 25 females were diagnosed with DGC and 2,832 were controls. Before imputation, we generated an MDS plot to evaluate the genetic heterogeneity between participants in the SNUBH2_AA cohort using the same method used in the GC_HC cohort (Supplementary Fig. S2B). For the GWAS analysis, owing to the small case-to-control ratio, we matched patients with GC with controls using their top five PC scores, ensuring that each patient was matched with five controls without replacement. This was accomplished using the Matching R package (v4.9–9) [22]. Then we conducted a conditional logistic analysis using the survival R package (v.3.4-0) [23], adjusting for sex and age.

Identity-by-descent (IBD) among all participants was estimated using plink with pruned common variants across cohorts, selected using the same options as those used for generating MDS plots, and visualized as a heatmap with the pheatmap R package (v1.0.12) [24] (Supplementary Fig. S2C).

### Gene-set analysis

We conducted gene-based tests using summary statistics from the GWAS results with two different methods. First, we conducted gene-based tests using MAGMA (v1.10) [25] with default options, as implemented in FUMA (v1.6.0) [26], accessible at https://fuma.ctglab.nl/. Second, we conducted tests using SPrediXcan (v0.7.5) [27] with stomach tissue as the target, modeled by MASHR. The MASHR-based model was constructed using the hg38 human reference genome. Therefore, before executing SPrediXcan, we updated the variant information in the summary statistics from hg19 to hg38 using the LiftOver tool.

### Mediation analysis

We performed a mediation analysis to identify the genes that mediated the effects of significant variants on GC risk, adjusting for the effects of sex, age, and the top four PC scores. This analysis was conducted using the mediation R package (v4.5.0) [28] with 10,000 bootstrap iterations. Gene expression levels in stomach tissues were estimated from imputed datasets using PrediXcan (v0.7.5) [29] and the MASHR-based model.

### Mendelian randomization study

Mendelian randomization (MR) studies have been conducted to investigate the causal relationship between GC risk and the traits associated with the significant variants according to the OpenGWAS (https://gwas.mrcieu.ac.uk/). Summary statistics for laboratory phenotypes, such as red blood cell (RBC), white blood cell (WBC), neutrophil, and platelet counts [30], serum triglyceride [30], albumin [30], non-albumin protein (NAP) [31], and total protein (TP) levels [32], albumin-to-globulin (A/G) ratio [31], and estimated glomerular filtration rate (eGFR) [31] were obtained from the BioBank Japan Project (BBJ) and are accessible at https://pheweb.jp/.

For the MR study, we employed both Generalized Summary-data-based Mendelian Randomization (GSMR) [33], implemented in Genome-wide Complex Trait Analysis (GCTA) (v1.94.1) [34], and Mendelian Randomization Pleiotropy RESidual Sum and Outlier (MR-PRESSO) R package (v1.0) [35], GSMR was conducted using the default settings. In the case of MR-PRESSO, we designated the ‘*OUTLIERtest*’ and ‘*DISTORTIONtest*’ options as TRUE and set the value of the ‘*NbDistribution*’ option to increment from 1,000 to 10,000. This ensured that instrumental variables (IVs) were significantly associated with exposure (*P* < 5 × 10^–8^), did not violate the HEterogeneity In Dependent Instruments (HEIDI)-outlier assumption (*P* value of the HEIDI-outlier filtering analysis > 0.01). Also, no evidence of horizontal pleiotropic variants among the IVs, according to MR-PRESSO, was observed.

In addition, we performed multivariable MR (MVMR) using MVMR R package (v0.3) [36] to estimate the direct effects of both gene expression and phenotype on GC risk. GWAS summary statistics for gene expression were derived from predicted expression values for each cohort (SNUBH and GC_HC) using PrediXcan, and these were combined with inverse variance weighting by METAL. Significant variants were clumped using plink (‘*–clump-p1 5e-8 –clump-p2 1 –clump-r2 0.05 –clump-kb 1000*’), with variant correlation estimated from GC_HC cohort. BBJ GWAS summary statistics for traits were also used. IVs met the criteria for strong instruments (F statistics > 10) and showed no evidence of horizontal pleiotropy based on *P* value of Cochran’s Q statistic (*P*_Het_ > 0.05). The ‘gencov’ option was set to zero due to different samples being used for estimating gene and trait effects on GC risk. The MVMR analysis was conducted using gene expression and only one phenotype at a time, as we could not determine overlapping samples across phenotypes from BBJ.

An MR mediation analysis was conducted to test whether the effect of the gene on GC risk was mediated by a specific phenotype. For this analysis, we estimated the causal effect using the inverse variance weighted (IVW) MR method, as in the MVMR approach, implemented in MendelianRandomization R package (v0.9.0) [37]. To assess the mediation effect, we performed a Sobel test [38] using MR analysis results from the relationships between the gene and each phenotype, as well as between each phenotype and GC risk.

### Heritability estimation

We estimated the heritability of each phenotype using the LD score (LDSC; v1.0.1) [39]. For this analysis, only variants present in HapMap3 were included, utilizing pre-computed LD scores of EAS ancestry by LDSC authors. The value was considered as zero if the estimated heritability value was negative.

In addition, the local heritability of GC risk and related phenotypes was estimated using LAVA R package (v0.1.0) [40] to assess whether estrogen metabolism influences these traits. Candidate estrogen-related genes were selected based on Rending et al*.*[41], including Catechol-O-Methyltransferase (*COMT*), Cytochrome P450 Family 1 Subfamily A Member 1 (*CYP1A1*), Cytochrome P450 Family 1 Subfamily B Member 1 (*CYP1B1*), Cytochrome P450 Family 19 Subfamily A Member 1 (*CYP19A1*), Estrogen Receptor 1 (*ESR1*), Glutathione S-Transferase Mu 1 (*GSTM1*), Glutathione S-Transferase Pi 1 (*GSTP1*), Glutathione S-Transferase Theta 1 (*GSTT1*), Hydroxysteroid 17-Beta Dehydrogenase 1 (*HSD17B1*), Sulfotransferase Family 1A Member 1 (*SULT1A1*), and UDP Glucuronosyltransferase Family 1 Member A1 (*UGT1A1*), and genes associated with significant variants identified in our study. Genomic loci were defined as the region of each gene, obtained from the GeneCards database (https://www.genecards.org/), including a 500 kb flanking region in either side. EAS samples from the 1000G were used as references.

### Analysis of known genes

We used the GWAS Catalog, accessible at https://www.ebi.ac.uk/gwas/, to identify genes mapped from variants significantly associated with GC (*P* < 5 × 10^–8^), excluding those associated with multiple cancers. For the selected genes, we evaluated their significance in relation to DGC risk and explored heterogeneity across sexes as predicted by SPrediXcan in stomach tissue. Cochran’s Q test was used to test for heterogeneity across sexes.

## Results

### rs762855 was significantly associated with the risk of DGC in females

**Fig. 1:**
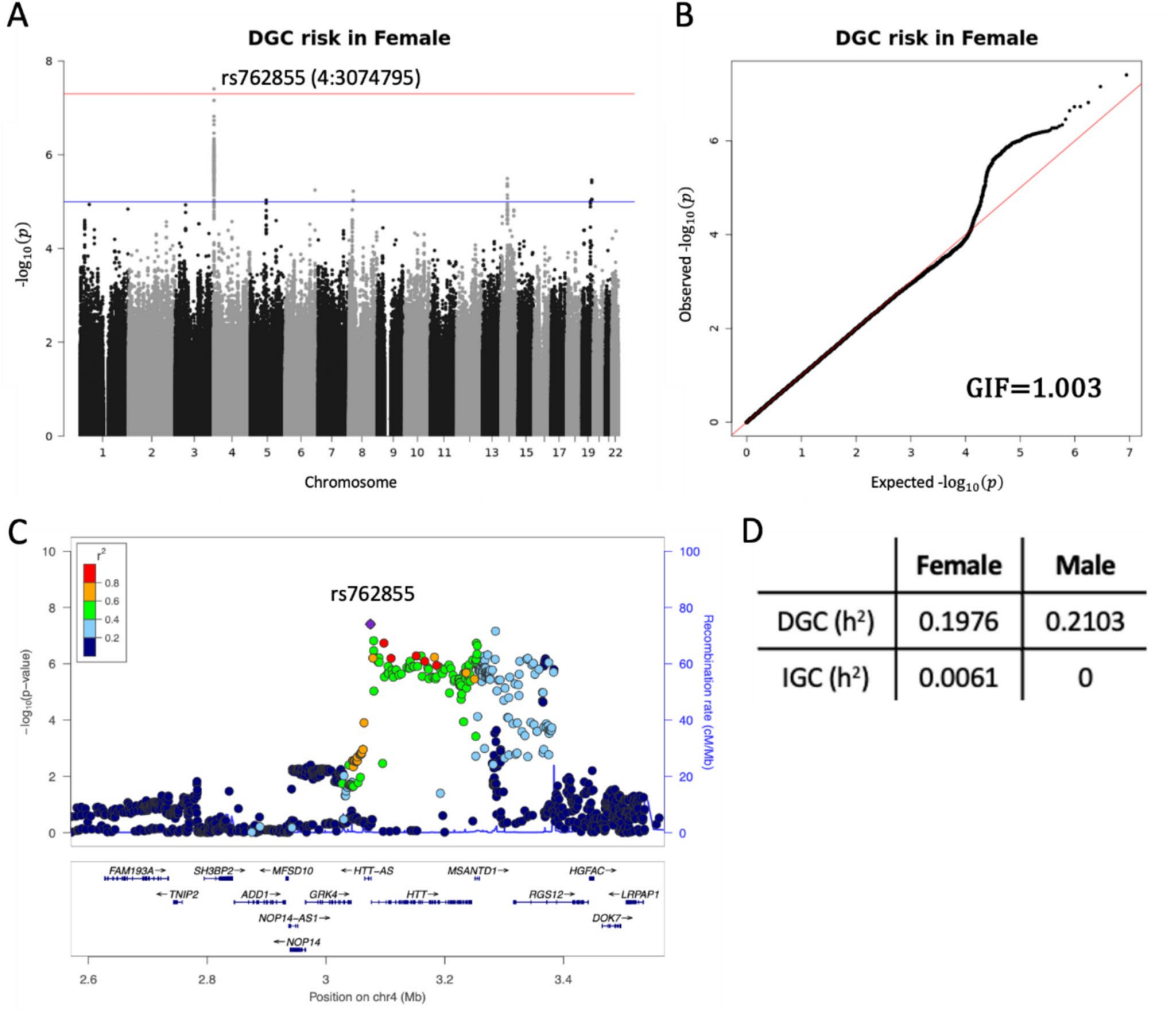
GWAS results for DGC risk in females and heritability of DGC and IGC risk across sexes. **A** Manhattan plot. The red and blue lines reflect significance levels of 5 × 10^−8^ and 1 × 10^−5^, respectively. **B** QQ plot. The x-axis and y-axis display expected and observed *P* values on a − log10 scale. **C** Locus zoom plot. This displays a 500-kb flanking region of the rs762855 locus. The square dot represents rs762855, and the circular dots represent other variants. The color indicates the correlation between rs762855 and other variants, ranging from red (high) to blue (low). Genes located near rs762855 are shown at the bottom. **D** Heritability estimates. This depicts the heritability of DGC and IGC risk across sexes. DGC, diffuse-type gastric cancer; IGC, intestinal-type gastric cancer; GIF, genomic inflation factor; *p*, *P* value; *r*^2^, correlation with rs762855; h^2^, heritability

The characteristics of all the cohort participants are summarized in Table 1. We conducted a GWAS to elucidate the susceptibility loci associated with the risk of DGC. The SNUBH and GC_HC cohorts were separately utilized, and their variants were tested using SAIGE. Subsequently, the summary statistics were combined using METAL with inverse-variant weighting. The meta-analysis revealed that rs762855 (chr4:3,074,795; hg19) was significantly associated with the risk of DGC in females (Fig. 1A). The risk allele for rs762855 is A, and its associated *P* value and odds ratio (OR) [95% confidence intervals (CIs)] were 3.91 × 10^–8^ and 1.758 [1.438–2.150], respectively (Table 2). The risk allele frequencies of rs762855 in the controls (0.367 and 0.409 in SNUBH and GC_HC, respectively) were similar to those of the Korean population reference (0.391) according to KRGDB in dbSNP (https://www.ncbi.nlm.nih.gov/snp/). These results were reliable, as indicated by the QQ plot and genomic inflation factor (GIF; 1.003, Fig. 1B), and the high imputation quality scores of the variant (0.906 and 0.881 in SNUBH and GC_HC, respectively). As demonstrated in Fig. 1C, variants that highly correlated with rs762855 tended to have smaller *P* values compared to those with low correlations and were located near the Myb/SANT DNA Binding Domain Containing 1 (*MSANTD1*) and Huntingtin (*HTT*) genes. According to the GeneCards database, rs762855 was located in the GH04J003073 GeneHancer region (chr4:3,074,528–3,080,471; hg19), whose targets include *MSANTD1* and *HTT*.

**Table 1:** Characteristics of participants in the entire cohorts

	SNUBH			GC_HC			SNUBH2_AA		
	Controls (*n* = 441)	GC cases (*n* = 527)	*P* value	Controls (n = 5,439)	GC cases (*n* = 794)	*P* value	Controls (*n* = 5,410)	GC cases (*n* = 101)	*P* value
Sex (female; n)	248 (56.2%)	164 (31.1%)	< 0.001	2,958 (54.4%)	267 (33.6%)	< 0.001	2,832 (52.3%)	44 (43.6%)	0.099
Age (mean ± SD; y)	53.5 ± 12.0	60.3 ± 11.4	< 0.001	55.6 ± 8.6	56.1 ± 11.7	0.259	51.5 ± 8.5	55.4 ± 14.5	8.68 $\times$ 10^–3^
*H. pylori* (positive; n)	307 (69.6%)	454 (86.1%)	< 0.001	NA	NA	NA	NA	NA	NA
Smoker (ever; n)	156 (35.4%)	327 (62.0%)	< 0.001	NA	NA	NA	NA	NA	NA
Lauren (n)									
Diffuse	NA	159 (30.2%)	NA	NA	389 (49.0%)	NA	NA	40 (39.6%)	NA
Intestinal	NA	303 (57.5%)	NA	NA	405 (51.0%)	NA	NA	49 (48.5%)	NA
Others	NA	65 (12.3%)	NA	NA	0 (0%)	NA	NA	12 (11.9%)	NA

*P* values were calculated using χ^2^-test or Student’s *t* test*. n* the number of participants, *y* Years, *SD* Standard deviation, *GC* Gastric cancer, *NA* Not available

**Table 2:**
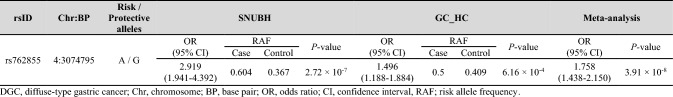
GWAS results for rs762855 regarding female DGC risk in each cohort and meta-analysis of the two cohorts (SNUBH and GC_HC)

The effects of the variant on DGC risk in females were consistent between pre- and post-menopausal groups (divided by age 50), with ORs of 1.681 and 1.691, respectively, and *P*_Het_ = 0.977 (Supplementary Table S1). In addition, the effects of the variant on DGC risk in females did not differ significantly across *H. pylori* status. In the overall female population, ORs were 3.094 and 3.678 in *H. pylori-*positive and -negative participants, respectively (*P*_Het_ = 0.811), although this could be only confirmed in the SNUBH cohort (Supplementary Table S2). Similarly, in pre-menopausal females, ORs were 6.258 and 2.705 in *H. pylori-*positive and -negative participants, respectively (*P*_Het_ = 0.508). However, in males, no significant variants were associated with the risk of DGC (Supplementary Fig. S3A). The statistics for rs762855 in males were as follows: *P* = 0.713, OR [95% CI]: 1.034 [0.866–1.235] (Supplementary Table S3).

The association between rs762855 and the risk of DGC in females was further validated using two independent cohorts, SNUBH2 and KOGES_Ansan/Ansung (SNUBH_AA). Although the number of female participants with DGC was relatively small (n = 25), the significant association was replicated (*P* = 0.0443, OR [95% CI]: 3.356 [1.031–10.92], Table 3). When conducting a meta-analysis with the results from all cohorts, the association became even more significant (*P* = 9.30 × 10^–9^, OR [95% CI]: 1.789 [1.467–2.182]). Furthermore, rs3135064 (chr4:3,285,389) and rs28820097 (chr4:3,097,495) were identified as significant, although they were not independent of rs762855 according to the LDpair with the EAS population (https://ldlink.nih.gov/?tab=ldpair) (R^2^ > 0.3, Supplementary Table S4).

**Table 3:**
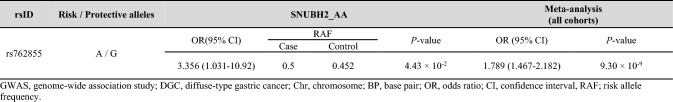
GWAS results of rs762855 in female DGC risk in the validation cohort and meta-analysis of all three cohorts (SNUBH, GC_HC, and SNUBH2_AA)

The heritability of DGC risk was similar between both sexes, with values of 0.1976 in females and 0.2103 in males (Fig. 1D). Conversely, IGC heritability was very low, with values of 0.0061 in females and 0 in males. Furthermore, no significant variants were associated with IGC risk in either females or males (Supplementary Fig. S3B and S3C).

Independent variants with *P* values less than 1 $\times$ 10^–5^ and R^2^ values less than 0.05 from GWASs across cancer types and sexes were summarized in Supplementary Table S5.

### *MSANTD1* is more frequently expressed in females with DGC than in controls and mediates the effect of rs762855 on DGC risk in females

**Fig. 2:**
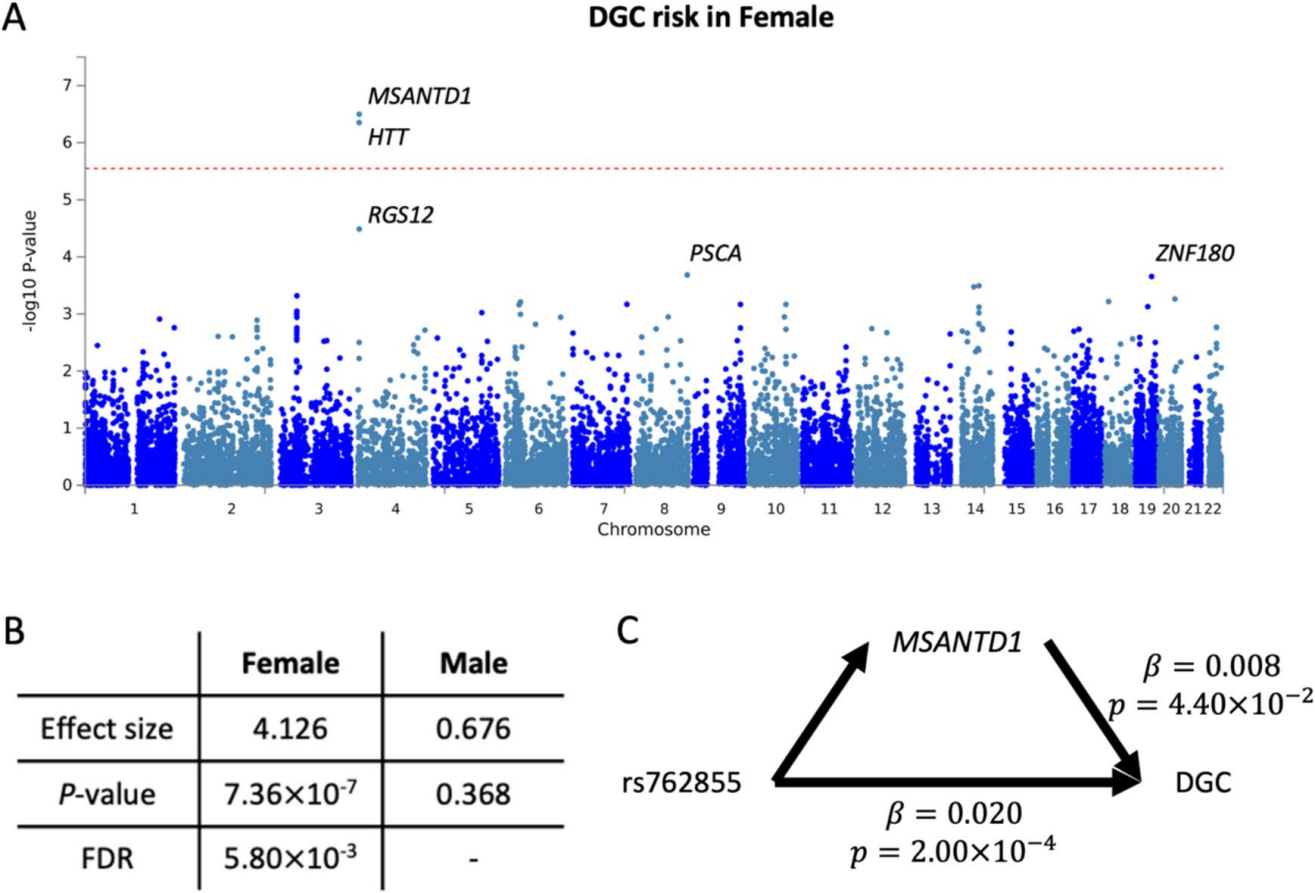
Gene-based test for DGC risk and mediation analysis. **A** Manhattan plot of gene-set analysis in females. The red dotted line reflects a Bonferroni-corrected *P* value of 0.05. The top five genes are denoted. **B** SPrediXcan results for *MSANTD1* according to sex. **C** Directed acyclic graph demonstrating that *MSANTD1* mediates the effect of rs762855 on the risk of DGC in females. DGC diffuse-type gastric cancer, *FDR* false discovery rate, *β* size of average causal mediation effect or average direct effect, *p P* value

We conducted a gene-set analysis using MAGMA implemented in FUMA with the results of the meta-analysis using the SNUBH and GC_HC cohorts. The analysis demonstrated that at Bonferroni-corrected *P* < 0.05, *MSANTD1* and *HTT* were the two genes most significantly associated with susceptibility to DGC in females (Fig. 2A). Furthermore, we performed a transcriptomic analysis using SPrediXcan. In stomach tissue, *MSANTD1* was predicted to be significantly and more highly expressed in DGC cases than in controls in females (*β* = 4.126, *P* = 7.36 × 10^−7^, false discovery rate (FDR) = 5.80 × 10^−3^) (Fig. 2B). However, no significant association was observed between DGC risk and the expression level of *MSANTD1* in males (*β* = 0.676 and *P* = 0.368). *HTT* could not be analyzed using SPrediXcan because its expression levels could not be predicted. However, according to the Eukaryotic Promoter Database (https://epd.expasy.org/epd/), several putative binding sites for the estrogen receptor 1 and estrogen receptor 2 genes, but not the androgen receptor gene, were identified as the most abundant promoter regions (HTT_1, Supplementary Fig. S4), while no information was available for *MSANTD1* in* Homo sapiens*.

Furthermore, mediation analysis demonstrated that *MSANTD1* mediates the effect of rs762855 on the risk of DGC in females, with an average causal mediation effect of *β* = 0.008 and *P* = 4.40 × 10^−2^ and an average direct effect of *β* = 0.020 and *P* = 2.00 × 10^−4^ (Fig. 2C). This indicated that rs762855 affects the risk of DGC partly through *MSANTD1*.

### Elevated globulin levels may increase the risk of DGC in females

**Fig. 3:**
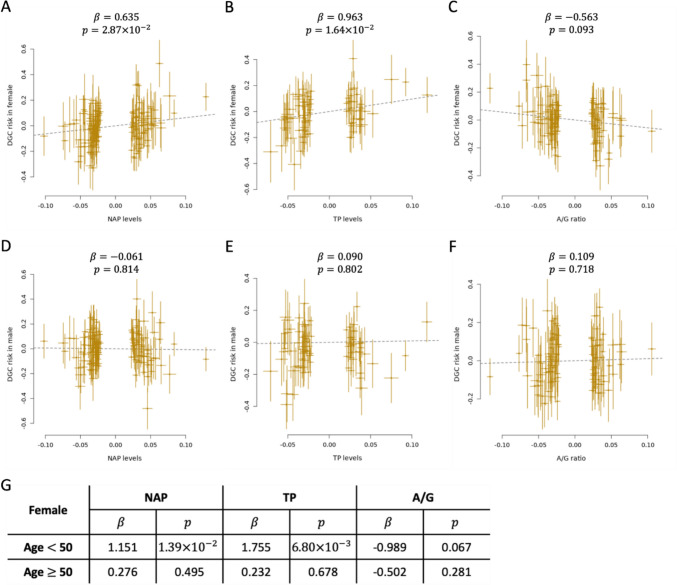
Mendelian randomization study. Causal relationships estimated by GSMR between DGC risk and **A** NAP levels in females, **B** TP levels in females, **C** A/G ratio in females, **D** NAP levels in males, **E** TP levels in males, or **F** A/G ratio in males. **G** Causal relationships estimated by GSMR between DGC risk and NAP, TP, or A/G in females across ages. *DGC* Diffuse-type gastric cancer, *NAP* non-albumin protein, *TP* total protein, A/G albumin-to-globulin ratio, *β* the effect size of a causal relationship; *p P* value

According to the OpenGWAS, rs762855 was associated with several traits including sex hormone-binding globulin (SHBG) and biomarkers (Supplementary Table S6). SHBG was not available in the BBJ; therefore, as alternatives, we investigated whether protein measurements including albumin, TP, NAP, and A/G ratio, as well as RBC, WBC, neutrophil, and platelet counts, serum triglyceride, and eGFR, were causally associated with the risk of DGC in both males and females using GSMR. The results showed that an increase in NAP levels led to an increased risk of DGC in females (*β* = 0.635 and *P* = 2.87 × 10^–2^; Fig. 3A). Elevated TP levels were associated with an increased risk of DGC in females (*β* = 0.963 and *P* = 1.64 × 10^–2^; Fig. 3B). Conversely, the causal relationship between the A/G ratio and DGC risk in females was not significant (*β* = -0.563 and *P* = 0.093, Fig. 3C). In males, however, no significant causal associations were noted between DGC risk and NAP levels (*β* = -0.061 and *P* = 0.814; Fig. 3D), TP levels (*β* = 0.090 and *P* = 0.802; Fig. 3E), or A/G ratio (*β* = 0.109 and *P* = 0.718; Fig. 3F). Interestingly, when we divided female participants into two groups based on their age (≥ 50 y or < 50 y), corresponding to post- and pre-menopausal statuses, respectively, the causal relationships between protein measurements and DGC risk became prominent in the pre-menopausal group only (Fig. 3G). Furthermore, MR analyses using MR-PRESSO presented results similar to those obtained using GSMR. However, a decrease in the A/G ratio was noted to significantly increase the risk of DGC in females (*β* = -0.659 and *P* = 3.21 × 10^–2^, Supplementary Table S7).

Furthermore, we conducted MVMR and MR mediation analyses to assess whether NAP levels, TP levels, or A/G ratio mediate the effect of *MSANTD1* on DGC risk in females. The MVMR results indicated that the direct effects of *MSANTD1* and each trait on DGC risk were similar to those observed in SPrediXcan and univariable MR analyses (Supplementary Table S8). The MR mediation analyses also showed no significant mediation effects of these traits (Supplementary Table S8).

Serum albumin levels and other biomarkers were not found to be causally related to the DGC risk in both sexes according to GSMR and MR-PRESSO (Supplementary Fig. S5), with exception of eGFR, which showed a significant relationship with DGC risk in male and by MR-PRESSO only (*β* = 1.645 and *P* = 3.85 × 10^–2^).

### rs11648430 is a susceptible variant for DGC only in pre-menopausal women

Given that DGC is more prevalent among young females [11] and that NAP or TP were identified as potential causal factors in pre-menopausal but not in post-menopausal females, we conducted separate GWAS analyses for pre- and post-menopausal females to elucidate genetic differences associated with DGC risk across these two groups.

GWAS were conducted in females, divided into pre- and post-menopausal groups, which might similarly categorize high- and low-estrogen groups, respectively. They were not only designed for MR but also aimed to explore genetic loci that may be associated with the risk of DGC. A meta-analysis of SNUBH and GC_HC datasets identified a locus that was significantly associated with the risk of DGC in the pre-menopausal group; its most significant variant was rs11648430 (chr16:29,022,557; hg19; *P* = 3.81 × 10^–8^ and OR [95% CI]: 3.003 [2.029–4.445], Supplementary Fig. S6A and Supplementary Table S9). However, this association could not be validated because of the limited number of female patients with DGC aged < 50 y in the validation cohort (n = 16). In addition, no genes were associated with the risk of DGC in the pre-menopausal group using MAGMA or SPrediXcan (in stomach tissue), and no phenotypes were significantly associated with rs11648430 using the LDtrait. Nevertheless, the expression levels of Tu Translation Elongation Factor, Mitochondrial (*TUFM*), Sulfotransferase Family 1A Member 2 (*SULT1A2*), and SPNS Lysolipid Transporter 1, Lysophospholipid (*SPNS1*) negatively correlated with the number of risk alleles (G) of rs11648430 in whole blood tissue according to the GTEx dataset (v8) (https://gtexportal.org/home/) [42] with *P* values of 3.06 × 10^–8^, 2.59 × 10^–5^, and 1.74 × 10^–4^, respectively (Supplementary Fig. S6B-D). In addition, rs11648430 is a spliced quantitative trait locus for Ataxin 2 Like (*ATXN2L*) and Eukaryotic Translation Initiation Factor 3 Subunit C (*EIF3C*) in whole blood tissue in the GTEx dataset. As the number of risk alleles for rs11648430 increased, more introns were excised from *ATXN2L* and fewer from *EIF3C* (Supplementary Fig. S6E-F). We extracted traits associated with rs11648430 from the OpenGWAS (Supplementary Table S10) and explored the potential causal relationship of RBC with DGC risk in the pre- and post-menopausal groups. However, both GSMR and MR-PRESSO indicated that RBC had no significant causal relationships with DGC risk in either group (Supplementary Fig. S7).

The rs11648430 was not associated with the risk of DGC in post-menopausal women (*P* = 0.257 and OR [95% CI]: 1.207 [0.872–1.671]) or men (*P* = 0.366 and OR [95% CI]: 1.104 [0.891–1.368]). Moreover, no variants were significantly associated with the risk of DGC in post-menopausal women (Supplementary Fig. S6G).

### Estrogen-associated genes influence NAP levels as well as DGC risk in females

**Fig. 4:**
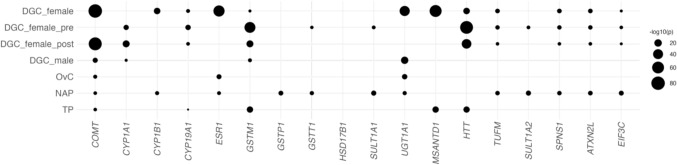
Local heritability. Local heritability was estimated for DGC risk (stratified by sex and menopausal status), ovarian cancer risk, non-albumin protein levels, and total protein levels within each genomic region, including a 500 kb flanking region around genes known to be associated with estrogen or identified as associated with DGC risk in females, using LAVA. The y-axis represents the stratified groups or traits, and the x-axis shows the gene names. Circle size indicates the significance level of non-zero heritability, represented as $-{\text{log}}_{10}(p)$. *DGC* diffuse-type gastric cancer, *DGC_female_pre* DGC risk in pre-menopausal females, *DGC_female_post* DGC risk in post-menopausal females, *OvC* ovarian cancer, *NAP* non-albumin protein, *TP* total protein, *p* *P* value

Next, we conducted local heritability estimation for DGC risk, NAP, TP, and ovarian cancer (OvC) across each genomic region of estrogen-associated genes (EAGs) and genes associated with DGC risk identified in our study. The analysis revealed that genes with significant non-zero heritability were much more prevalent in females than in males, and interestingly, several genes influenced NAP levels as well as DGC risk in females (Fig. 4). *CYP1B1* and *ESR1* affected NAP levels and DGC risk exclusively in females, while *GSTT1*, *SULT1A1*, and *SULT1A2* influenced NAP levels and DGC risk specifically in pre-menopausal females.

The analysis also indicated that *HTT* as well as *MSANTD1* were associated with both TP levels and DGC risk in females. In addition, *TUFM*, *SPNS1*, *ATXN2L*, and *EIF3C*, which were identified as being associated with DGC risk in pre-menopausal females, may also impact DGC risk in post-menopausal females. The risk of OvC, however, was not influenced by the genes identified to be associated with DGC risk in females.

## Association of **PRKAA1** and *TTC33* with DGC risk varies according to sex

Among the 40 genes associated with the risk of GC based on the GWAS Catalog, 12 could be predicted by SPrediXcan. We tested whether the association between these genes and DGC risk differed according to sex. The analysis revealed that Protein Kinase AMP-Activated Catalytic Subunit Alpha 1 (*PRKAA1*) and Tetratricopeptide Repeat Domain 33 (*TTC33*) exhibited heterogeneity between the sexes. The *P*_Het_ were 1.60 × 10^−2^ and 3.49 × 10^−2^ for *PRKAA1* and *TTC33*, respectively (Table 4). The expression level of *PRKAA1* was not significantly associated with the risk of DGC in females (*β* = − 0.367 and *P* = 0.737) but was significantly associated in males (*β* = $-$ 3.629 and *P* = 2.90 × 10^−4^). Similarly, the association between *TTC33* expression levels and DGC risk was significant only in males (*β* = 2.652 and *P* = 1.13 × 10^−2^), but not in females (*β* = − 0.077 and *P* = 0.946).

**Table 4:** SPrediXcan results testing the association between DGC risk and reported genes across sexes

Gene	Female		Male		Q	*P*_Het_
	$\beta$	*P*	$\beta$	*P*		
*PRKAA1*	−0.367	0.737	−3.629	2.90 $\times$ 10^–4^	5.805	1.60 $\times$ 10^–2^
*TTC33*	−0.077	0.946	2.652	1.14 $\times$ 10^–2^	4.449	3.49 $\times$ 10^–2^
*UNC5CL*	1.784	0.141	0.267	0.810	1.544	0.214
*HLA-C*	0.885	0.289	0.017	0.966	0.101	0.751
*JRK*	0.653	0.695	0.542	0.714	0.015	0.903
*ABO*	−0.273	0.166	−0.513	2.57 $\times$ 10^–3^	0.001	0.975
*HLA-A*	0.724	2.89 $\times$ 10^–2^	0.645	2.91 $\times$ 10^–2^	0.000	0.986
*PSCA*	0.311	1.07 $\times$ 10^–4^	0.146	4.13 $\times$ 10^–2^	0.000	0.993
*LY6K*	0.334	$2.40\times$ 10^–2^	0.249	0.055	0.000	0.993
*NPIPB2*	−0.115	0.307	−0.102	0.320	0.000	0.999
*LMNA*	−27.274	0.840	10.154	0.934	1.15 $\times$ 10^7^	0
*GON4L*	2.702	0.493	−5.120	0.144	419.1	3.85 $\times$ 10^–93^

*β* effect size, *P P* value, *Q* Cochran’s Q statistic, *P*_Het_* P* value of Cochran’s Q test

*PSCA* demonstrated a significant association with the risk of DGC in both sexes ($\beta$= 0.311 and *P* = 1.07 × 10^−4^ in females; *β* = 0.146 and *P* = 4.13 × 10^−2^ in males) and was not found to be heterogeneous across sexes (*P*_Het_ = 0.993). In contrast, Lamin A/C (*LMNA*) and Gon-4 Like (*GON4L*) demonstrated significant heterogeneity across sexes (*P*_Het_ < 0.05), although their associations were not significant for each sex.

## Discussion

Our results suggest that the rs762855 SNP modulates the risk of DGC in Korean females. Genetic variation in rs762855 reportedly modulates the risk of Huntington’s disease [43]. However, to the best of our knowledge, no previous studies have demonstrated that mutations in rs762855 affect the risk of developing GC. Thus, in our study, we performed a gene-set analysis, and we found that *MSANTD1* and *HTT* were the two genes most significantly associated with susceptibility to DGC in females.

The transcriptomic analysis confirmed that in stomach tissue, *MSANTD1* expression was significantly higher in women with DGC than in controls. Moreover, the mediation analysis indicated that rs762855 affected the risk of DGC partly through *MSANTD1*. Unfortunately, research on *MSANTD1* is very scarce, rendering it difficult to explain the mechanism by which *MSANTD1* increases the risk of DGC in females. Nevertheless, one study linked *MSANTD3* to salivary gland acinic cell carcinoma [44]. In addition, another study reported Myb as an oncoprotein [45]. Notably, *MSANTD1* is most enriched in fibroblasts within stomach tissue, as reported by the Human Protein Atlas (https://www.proteinatlas.org/). Harmonizome 3.0 further identifies the transcription factor STAT3 as a potential regulator of *MSANTD1* (https://maayanlab.cloud/Harmonizome/). STAT3 plays a critical role in cancer by promoting tumor growth and immune evasion within the tumor microenvironment (TME), particularly through its regulation of cancer-associated fibroblasts [46, 47]. This interaction suggests that *MSANTD1* may influence the TME in DGC through its regulation by STAT3. Further studies are required to explore the role of *MSANTD1* in the context of DGC risk.

Next, MR analyses indicated that serum protein levels might be causally related to DGC risk in females independent from *MSANTD1*. First, the A/G ratio showed a significant direct causal effect on DGC risk in females, with no mediation effect between *MSANTD1* and DGC risk. Previous studies reported that low preoperative serum albumin levels were associated with a worse survival outcome; thus, it may act as a surrogate marker in predicting the prognosis of patients with GC [48]. The albumin and A/G ratio may reflect the nutritional status and severity of disease in cancer patients [49, 50]. Interestingly, one study clearly suggested that a low A/G ratio was associated with cancer incidence and mortality in the general population [51, 52]. In our study, serum protein levels, particularly NAP levels, were significantly associated with an increased risk of DGC in pre-menopausal women; however, no such significant results were found in men. One hypothesis is that NAP may reflect not only immunoglobulins related to chronic inflammation but also the levels of sex hormones or SHBG. Notably, an increase in serum SHBG levels has been associated with an increased risk of GC in men [53, 54]. Thus, our findings should be investigated further in future studies.

Other novel findings in our study were that the rs11648430 SNP was found to be significantly associated with DGC risk in females aged < 50 y but not in men or in females aged ≥ 50 y. rs11648480 was significantly associated with mRNA expression of *TUFM*, *SULT1A2*, and *SPNS1*. Especially, *TUFM* has been linked to tumors in the digestive system, including GC [55]. Furthermore, the complex formed by its product and the product encoded by the NOD-Like Receptor X1 (*NLRX1*) gene, which reduces inflammation in female mice but not in male mice, has been reported to regulate type 1 interferon and autophagy [56].

Considering that DGC is prevalent in pre-menopausal women, this SNP may have clinical implications. Nevertheless, since we did not find any significant phenotypes which connect rs11648430 and DGC risk, further mechanistic studies are needed.

Local heritability analysis suggested that estrogen might influence both female DGC and OvC risks, the latter being a well-known estrogen-associated cancer [57, 58]. However, LDSC analysis shows no significant genetic correlation between the two cancers (${r}_{g}=-0.217$ and *P* $=0.772$), and the genes identified in this study did not appear to influence the susceptibility to OvC. This implies that estrogen-related mechanisms may affect the two cancers differently, although some genomic regions, including *COMT*, *ESR1*, and *UGT1A1*, impact both the risks of female DGC and OvC. Interestingly, these genes also influence serum NAP levels.

As previously mentioned, sex differences play a role in the etiology of GC. These differences may be genetically explained. In our study, additional SPrediXcan analysis revealed that *PRKAA1* and *TTC33* were associated with DGC incidence in males, while *PSCA* appears to increase DGC risk in both males and females. However, detailed analysis of these genes, including relationship with male hormone metabolism, remains beyond the scope of our study. Further research is needed to explore the genetic factors underlying sex disparities in GC.

**Fig. 5:**
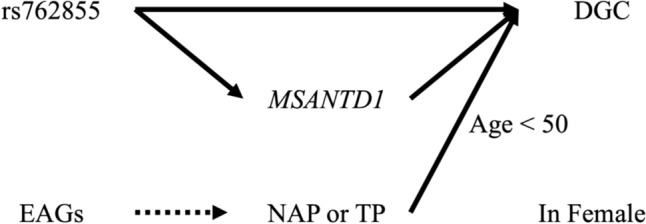
Directed acyclic graph of risk factors for DGC in female. The graph illustrates potential pathways linking estrogen-associated genes (EAGs), non-albumin protein (NAP), and total protein (TP) to DGC risk in females. Dashed line represents the path from EAGs to NAP or TP, indicating that we did not assess whether EAGs influence DGC risk through NAP or TP. However, EAGs are shown to influence both NAP (or TP) and DGC risk in females. *DGC* Diffuse-type gastric cancer, *EAG* Estrogen-associated gene, *NAP* Non-albumin protein, *TP* Total protein

In Fig. 5, we propose a directed acyclic graph as a summary to illustrate the risk factors for DGC in females and their potential pathways. However, the present study has several limitations. First, the sample size of patients with GC was relatively small for the subgroup analysis. Second, although both the SNUBH and SNUBH2 cohorts contained information on *H. pylori* status, the GC_HC and KoGES_Ansan/Ansung datasets did not contain information on *H. pylori* status. Third, the rs2671655, which demonstrated significant association in *H. pylori*-positive individuals [12], was not reproduced in this study (a meta-analysis of SNUBH and GC_HC cohorts, GC risk regardless of *H. pylori* infection: *β* = 0.068, OR [95% CI] = 1.070 [0.953–1.201], *P* = 0.251). Due to the lack of information on *H. pylori* infection in the GC_HC cohort, we were unable to perform a subgroup analysis according to *H. pylori* status.

In conclusion, we identified rs762855, *MSANTD1*, and serum NAP levels as potential risk factors for developing DGC in Korean females. Further research is necessary to explore the link between the rs762855 and NAP, as well as the underlying mechanisms by which they increase the risk of female DGC.

## Supplementary Information

Below is the link to the electronic supplementary material.

Supplementary file1 (PDF 1489 KB)

Supplementary file2 (PDF 390 KB)

## Data Availability

The datasets used and/or analyzed during the current study are available from the corresponding author.

## Abbreviations

*GC*: Gastric cancer
*H. pylori*: *Helicobacter pylori*
*IGC*: Intestinal-type gastric cancer
*AG*: Atrophic gastritis
*IM*: Intestinal metaplasia
*DGC*: Diffuse-type gastric cancer
*GWAS*: Genome-wide association study
*SNP*: Single nucleotide polymorphism
*PSCA*: Prostate Stem Cell Antigen
*MUC1*: Mucin 1, Cell Surface Associated
*CUX2*: Cut Like Homeobox 2
*ZNF652*: Zinc Finger Protein 652
*SPOP*: Speckle Type BTB/POZ Protein
*SNUBH*: Seoul National University Bundang Hospital
*QC*: Quality control
*NBK*: National Biobank of Korea
*KoGES*: Korean Genome and Epidemiology Study
*KoGES_HEXA*: KoGES health examinee
*KoGES_CAVAS*: KoGES cardiovascular disease association study
*GC_HC*: The combined cohort comprising GC, KoGES_HEXA, and KoGES_CAVAS cohorts from NBK
*MAF*: Minor allele frequency
*MDS*: Multi-dimensional scaling
*PC*: Principal component
*QQ*: Quantile–quantile
*SNUBH2_AA*: The combined cohort comprising SNUBH2 and KoGES_Ansang/Ansung cohorts
*IBD*: Identity-by-descent
*MSANTD1*: Myb/SANT DNA Binding Domain Containing 1
*MR*: Mendelian randomization
*Alb*: Albumin
*NAP*: Non-albumin protein
*TP*: Total protein
*A/G*: Albumin-to-globulin ratio
*NEU*: Neutrophil
*WBC*: White blood cell
*PLT*: Platelet count
*TG*: Triglyceride
*eGFR*: Estimated glomerular filtration rate
*RBC*: Red blood cell count
*BBJ*: BioBank Japan project
*GSMR*: Generalized summary-data-based mendelian randomization
*GCTA*: Genome-wide complex trait analysis
*MR-PRESSO*: Mendelian randomization pleiotropy residual sum and outlier
*IV*: Instrumental variable
*HEIDI*: Heterogeneity in dependent instruments
*MVMR*: Multivariable MR
*IVW*: Inverse variance weighted
*EAS*: East Asian
*COMT*: Catechol-O-Methyltransferase
*CYP1A1*: Cytochrome P450 Family 1 Subfamily A Member 1
*CYP1B1*: Cytochrome P450 Family 1 Subfamily B Member 1
*CYP19A1*: Cytochrome P450 Family 19 Subfamily A Member 1
*ESR1*: Estrogen Receptor 1
*GSTM1*: Glutathione S-Transferase Mu 1
*GSTP1*: Glutathione S-Transferase Pi 1
*GSTT1*: Glutathione S-Transferase Theta 1
*HSD17B1*: Hydroxysteroid 17-Beta Dehydrogenase 1
*SULT1A1*: Sulfotransferase Family 1A Member 1
*UGT1A1*: UDP Glucuronosyltransferase Family 1 Member A1
*OR*: Odds ratio
*CI*: Confidence interval
*HTT*: Huntingtin
*P*_Het_: *P* value of Cochran’s Q test
*FDR*: False discovery rate
*SHBG*: Sex hormone-binding protein
*TUFM*: Tu Translation Elongation Factor, Mitochondrial
*SULT1A2*: Sulfotransferase Family 1A Member 2
*SPNS1*: SPNS Lysolipid Transporter 1, Lysophospholipid
*ATXN2L*: Ataxin 2 Like
*OvC*: Ovarian cancer
*EAG*: Estrogen-associated gene
*PRKAA1*: Protein Kinase AMP-Activated Catalytic Subunit Alpha 1
*TTC33*: Tetratricopeptide Repeat Domain 33
*EIF3C*: Eukaryotic Translation Initiation Factor 3 Subunit C
*LMNA*: Lamin A/C
*GON4L*: Gon-4 Like
*TME*: Tumor microenvironment
*NLRX1*: NOD-Like Receptor X1
