# Supplementary material for: rs762855 single nucleotide polymorphism modulates the risk for diffuse-type gastric cancer in females: a genome-wide association study in the Korean population
Source: Gastric Cancer. 2025 Jan 25;28(2):145–59. doi: 10.1007/s10120-024-01575-6 (PMC11842433; doi:10.1007/s10120-024-01575-6)
Supplement: Supplementary file 1 — Supplementary file1 (PDF 1489 KB) [file 10120_2024_1575_MOESM1_ESM.pdf]

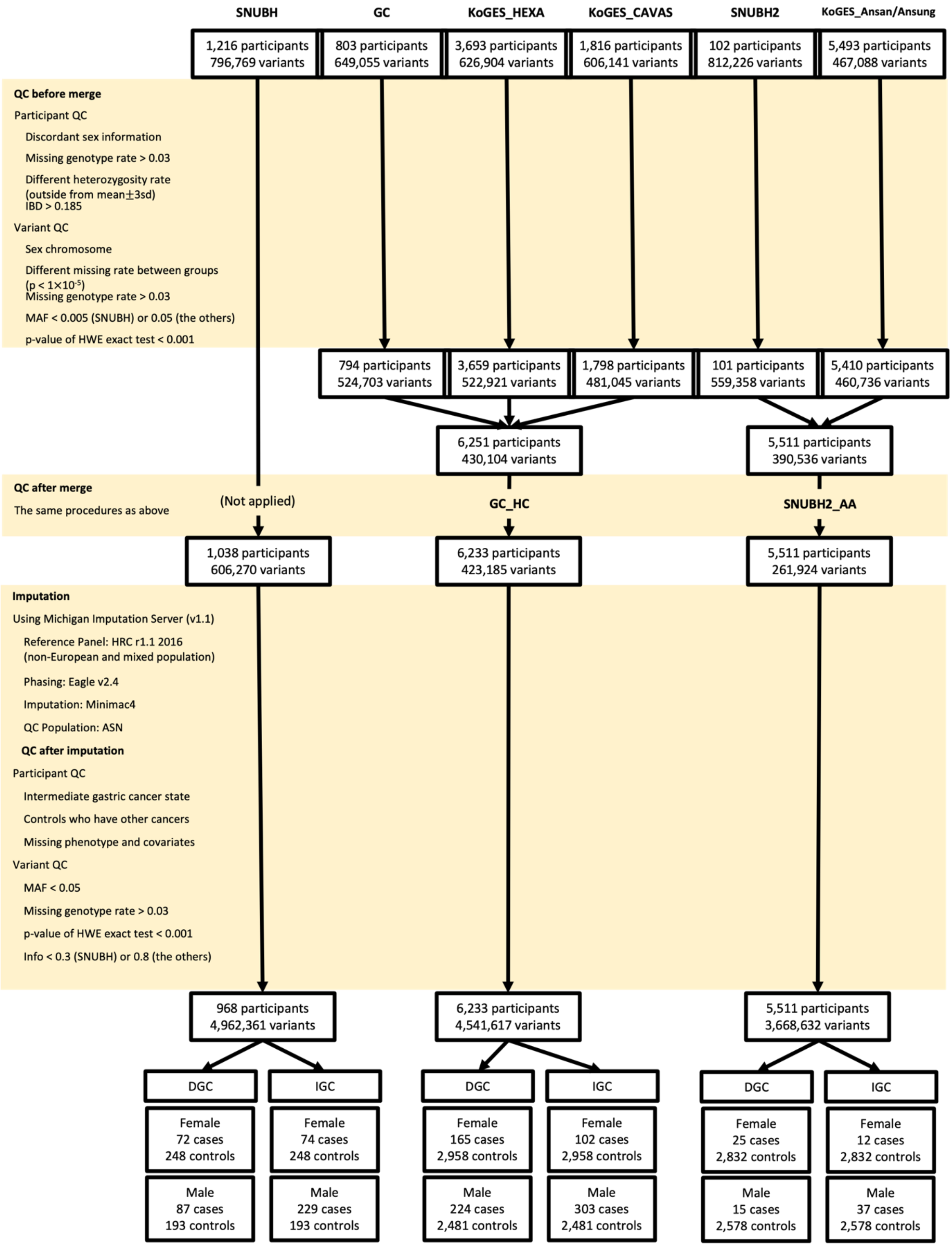

Supplementary Figure S1. Workflow of QC and imputation procedures. sd, standard deviation; IBD, identity by descent; MAF, minor allele frequency; HWE, Hardy-Weinberg equilibrium; *p*, p-value; DGC, diffuse-type gastric cancer; IGC, intestinal-type gastric cancer.

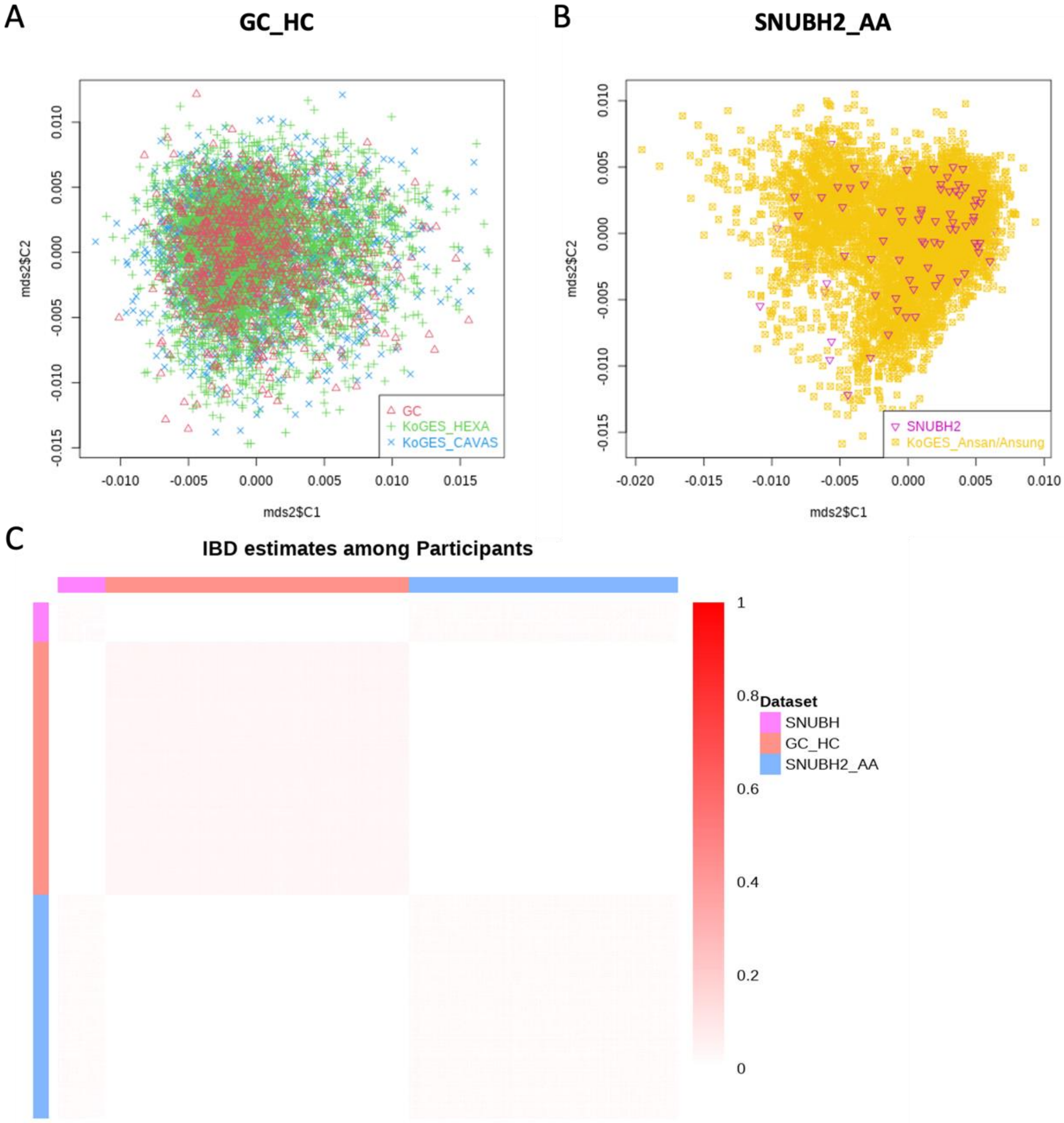

Supplementary Figure S2. MDS plots of participants in (A) GC_HC and (B) SNUBH2_AA. Colors and shapes of dots are based on their respective original cohorts. (C) Heatmap of Identity-by-descent (IBD) estimates among all participants.

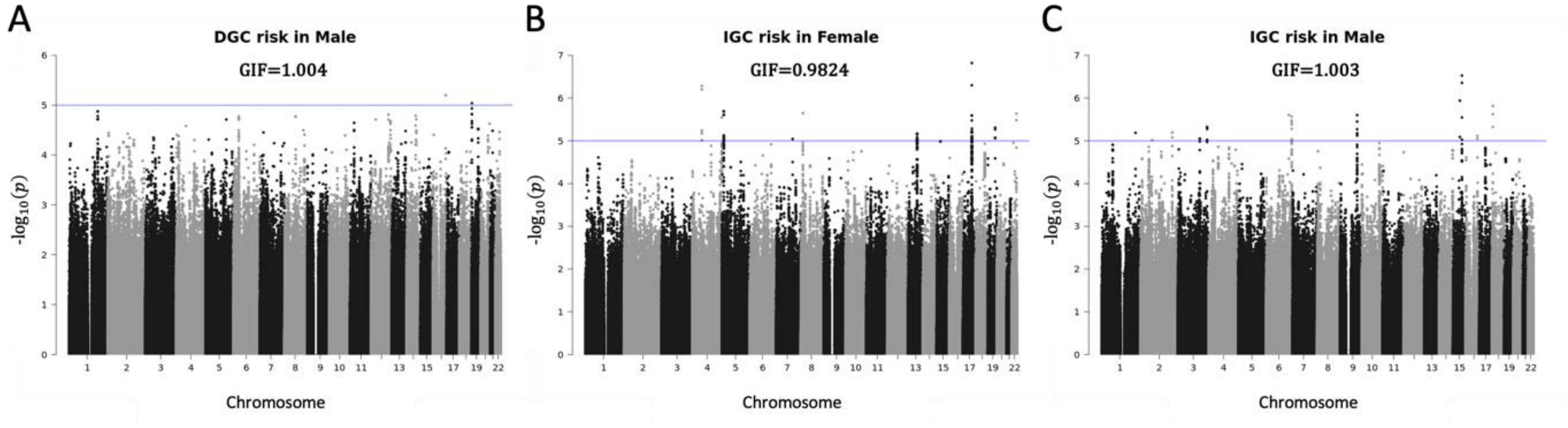

Supplementary Figure S3. Manhattan plots of GWAS for (A) DGC risk in males, (B) IGC risk in females, and (C) IGC risk in males. DGC, diffuse-type gastric cancer; IGC, intestinal-type gastric cancer; GIF, genomic inflation factor; *p*, *p*-value.

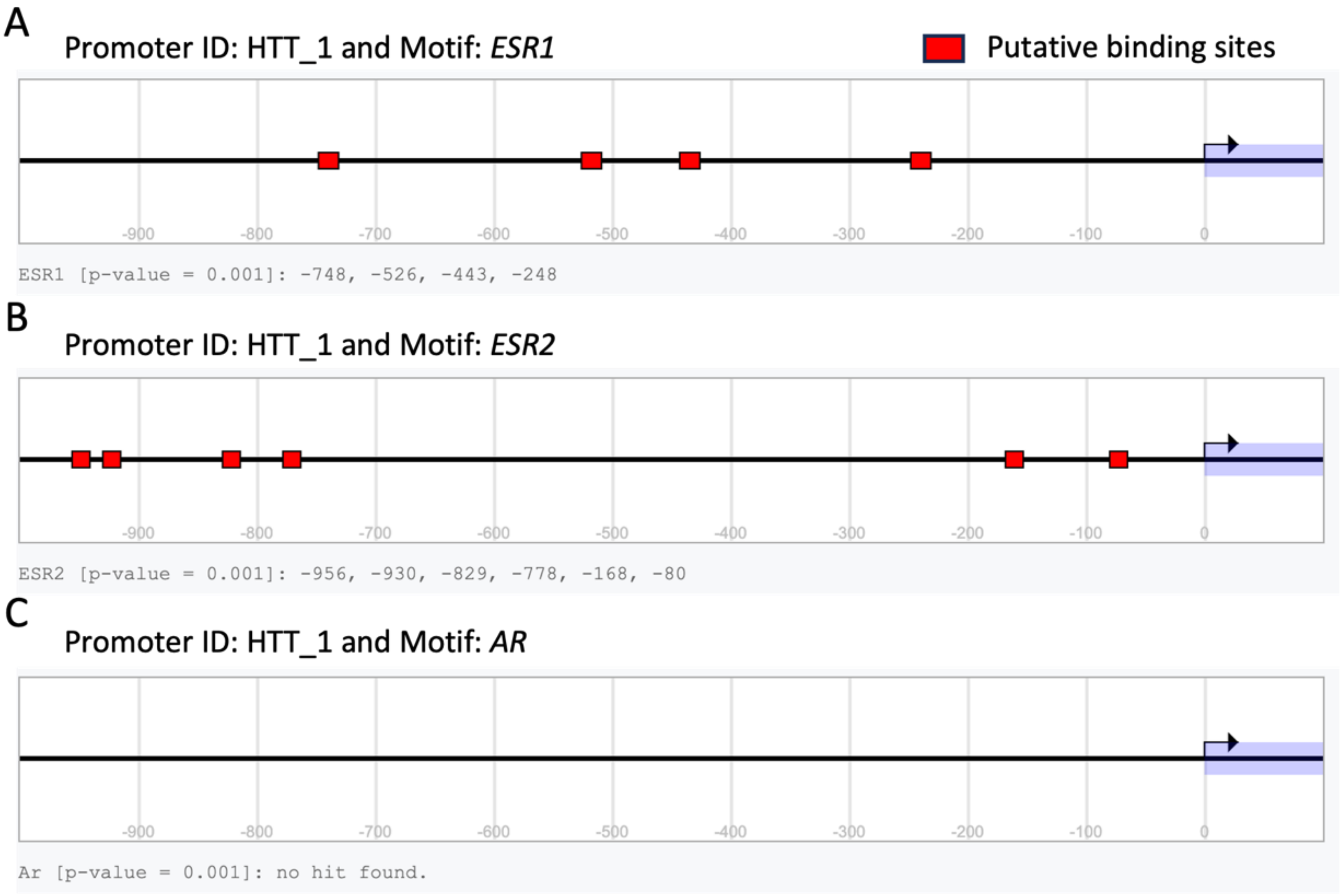

Supplementary Figure S4. Putative bindings sites in the promoter of *HTT* for (A) *ESR1*, (B) *ESR2*, and (C) *AR*.

A

$\beta = 0.277$
$p = 0.643$

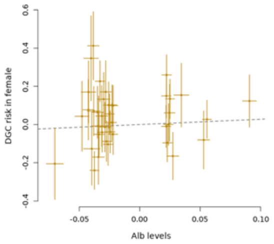

B

$\beta = 0.324$
$p = 0.544$

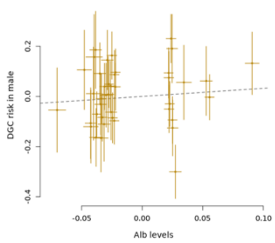

C

MR-PRESSO	Alb	
	β	p
Female	0.220	0.722
Male	0.247	0.633

D

$\beta = 0.438$
$p = 0.292$

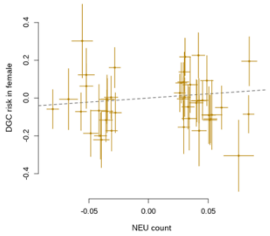

E

$\beta = 0.058$
$p = 0.876$

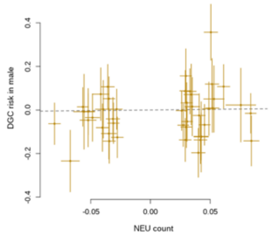

F

MR-PRESSO	NEU	
	β	p
Female	0.385	0.348
Male	0.327	0.490

G

$\beta = 0.671$
$p = 0.079$

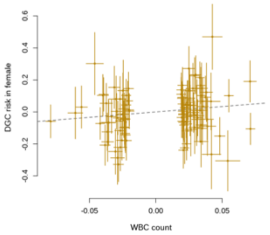

H

$\beta = -0.018$
$p = 0.958$

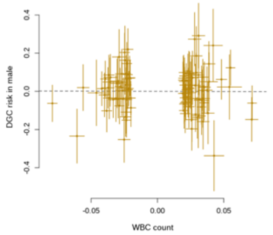

I

MR-PRESSO	WBC	
	β	p
Female	0.372	0.056
Male	0.286	0.973

J

$\beta = 0.089$
$p = 0.723$

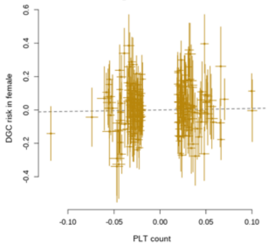

K

$\beta = -0.297$
$p = 0.188$

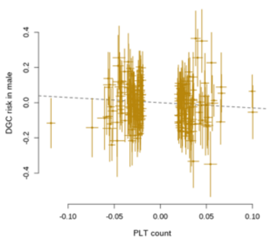

L

MR-PRESSO	PLT	
	β	p
Female	0.018	0.939
Male	0.206	0.408

M

$\beta = -0.230$
$p = 0.400$

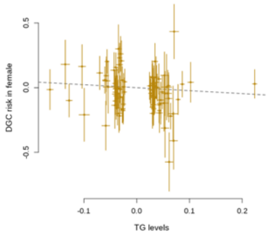

N

$\beta = 0.020$
$p = 0.936$

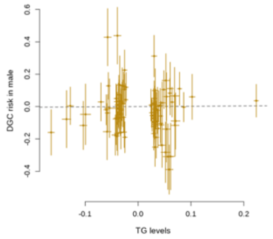

O

MR-PRESSO	TG	
	β	p
Female	-0.372	0.127
Male	-0.049	0.828

P

$\beta = 1.193$
$p = 0.166$

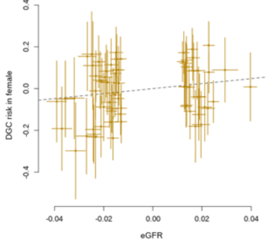

Q

$\beta = 1.247$
$p = 0.110$

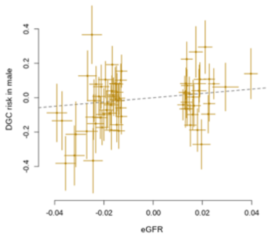

R

MR-PRESSO	eGFR	
	β	p
Female	1.403	0.068
Male	1.645	3.85×10^{-2}

Supplementary Figure S5. Mendelian randomization study of DGC risk and albumin (Alb) levels, neutrophil (NEU) count, white blood cell (WBC) count, platelet (PLT) count, triglyceride (TG) levels, or estimated glomerular filtration rate (eGFR). Causal relationships estimated by GSMR between Alb levels and DGC risk in (A) females and (B) males, and (C) causal relationship estimated by MR-PRESSO between DGC risk and Alb levels in females and males, respectively. Causal relationships between NEU count and DGC risk in (D) females and (E) males were estimated by GSMR, and (F) MR-PRESSO. Causal relationships between WBC count and DGC risk in (G) females and (H) males were estimated by GSMR, and (I) MR-PRESSO. Causal relationships between PLT count and DGC risk in (J) females and (K) males were estimated by GSMR, and (L) MR-PRESSO. Causal relationships between TG levels and DGC risk in (M) females and (N) males were estimated by GSMR, and (O) MR-PRESSO. Causal relationships between eGFR count and DGC risk in (P) females and (Q) males were estimated by GSMR, and (R) MR-PRESSO. DGC, diffuse type gastric cancer; Alb, albumin; NEU, neutrophil; WBC, white blood cell; PLT, platelet; TG, triglyceride; eGFR, estimated glomerular filtration rate; β, effect size of causal relationship; p, p-value.

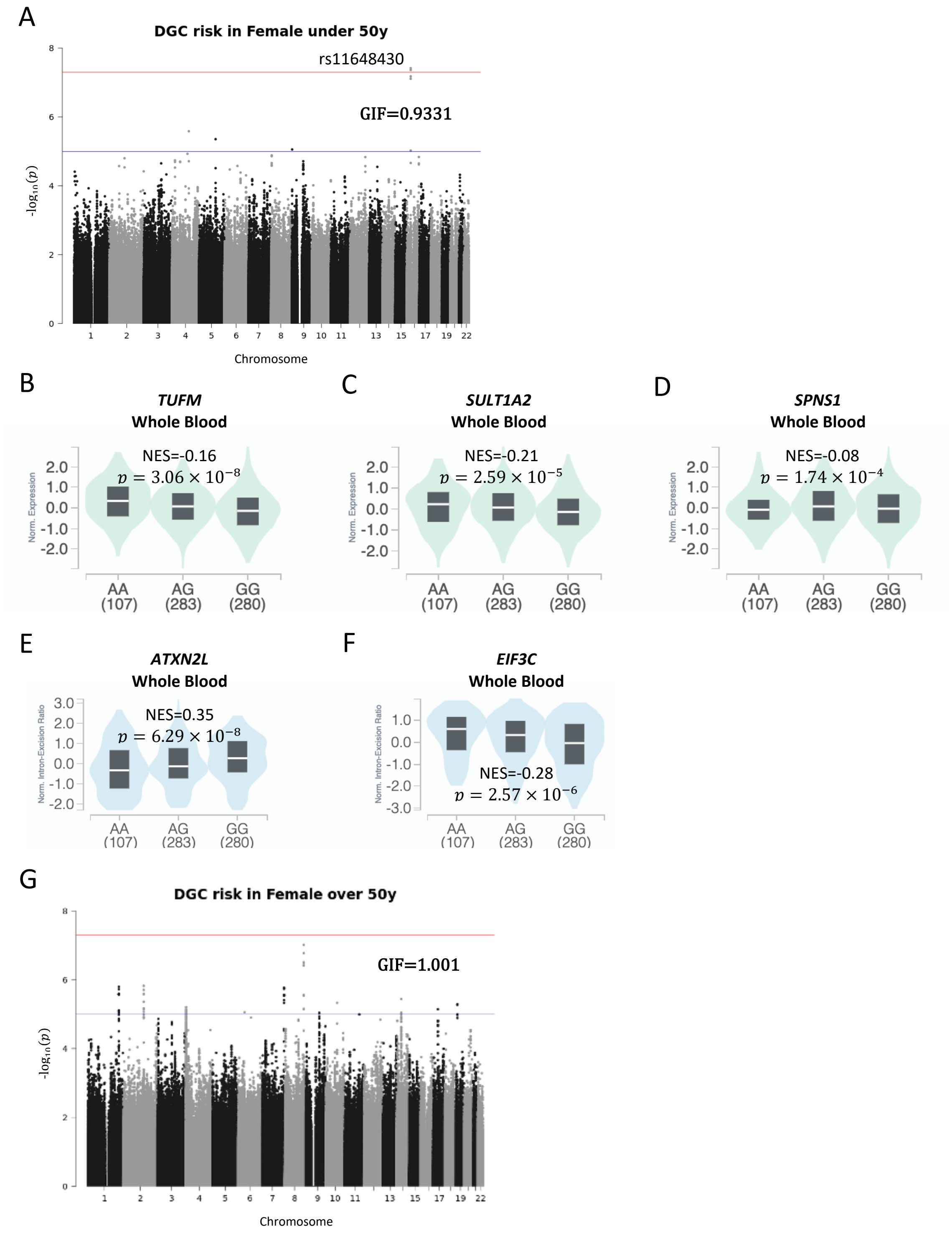

Supplementary Figure S6. Subgroup analysis in females according to age. (A) Manhattan plot of DGC risk in females under 50 years old. Box plot of expression levels of (B) *TUFM*, (C) *SULT1A2*, and (D) *SPNS1* and intron-excision ratio of (E) *ATXN2L*, and (F) *EIF3C* according to the number of risk alleles of rs11648430 in whole blood tissue. (G) Manhattan plot of GWAS results for DGC risk in females 50 years old or order. DGC, diffuse type gastric cancer; GIF, genomic inflation factor; *p*, p-value; 50y; 50 years old; NES, normalized effect size.

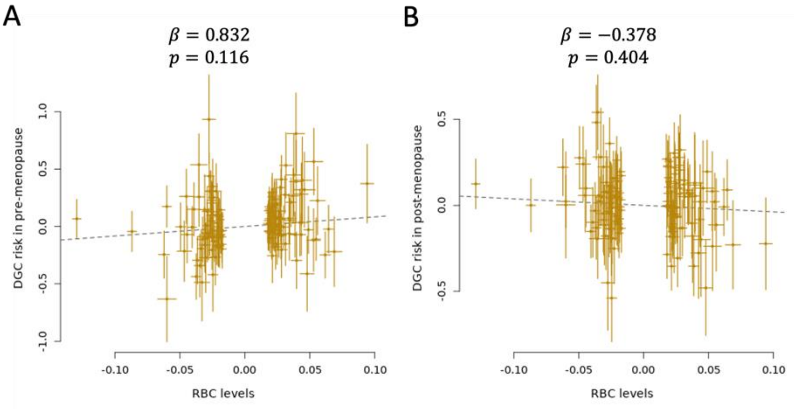

C

MR-PRESSO	RBC	
	β	p
**Pre**	0.867	0.074
**Post**	-0.417	0.313

Supplementary Figure S7. Mendelian randomization study of DGC risk and red blood cell (RBC) count in pre- and post-menopausal groups. Causal relationships between RBC and DGC risk in (A) pre- and (B) post-menopausal groups estimated by GSMR, and (C) by MR-PRESSO. DGC, diffuse type gastric cancer; RBC, red blood cell count; β, effect size of causal relationship; p, p-value.
