# Supplementary material for: rs762855 single nucleotide polymorphism modulates the risk for diffuse-type gastric cancer in females: a genome-wide association study in the Korean population
Source: Gastric Cancer. 2025 Jan 25;28(2):145–59. doi: 10.1007/s10120-024-01575-6 (PMC11842433; doi:10.1007/s10120-024-01575-6)
Supplement: Supplementary file 2 — Supplementary file2 (PDF 390 KB) [file 10120_2024_1575_MOESM2_ESM.pdf]

Supplementary Table S1. Association results of rs762855 with DGC risk in female according to age for each cohort and meta-analysis.

rsID	Chr:BP	Risk / Protective alleles	Groups	SNUBH			GC_HC			Metaanalysis	
				OR (95% CI)	RAF (Case / Control)	*P*-value	OR (95% CI)	RAF (Case / Control)	*P*-value	OR (95% CI)	*P*-value
rs762855	4:3074795	A / G	Age $<$ 50	3.574 (1.797-7.107)	0.688 / 0.379	2.83×10^{-4}	1.371 (0.955-1.968)	0.476 / 0.4	0.0869	1.681 (1.221-2.314)	1.50×10^{-3}
			Age $\geq$ 50	2.190 (1.254-3.825)	0.538 / 0.360	5.84×10^{-3}	1.556 (1.131-2.140)	0.524 / 0.413	6.55×10^{-3}	1.691 (1.282-2.232)	2.03×10^{-4}

Chr, chromosome; BP, base pair; OR, odds ratio; CI, confidence interval, RAF; risk allele frequency.

Supplementary Table S2. GWAS results of rs762855 DGC risk in females, stratified by *H. pylori*-infection status and age group in SNUBH cohort.

rsID	Chr:BP	Risk / Protective alleles		*H. pylori*-positive				*H. pylori*-negative				Heterogeneity	
				OR (95% CI)	RAF (*N*) Case	RAF (*N*) Control	*P*-value	OR (95% CI)	RAF (*N*) Case	RAF (*N*) Control	*P*-value	Q	*P*-value
rs762855	4:3074795	A / G	Females of all ages	3.094 (1.964-4.876)	0.609 (64)	0.380 (183)	1.12×10^{-6}	3.678 (0.960-14.09)	0.563 (8)	0.331 (65)	0.057	0.057	0.811
			Females < 50 y	6.258 (2.361-16.59)	0.714 (28)	0.371 (62)	2.27×10^{-4}	2.705 (0.276-27.55)	0.5 (4)	0.4 (25)	0.393	0.438	0.508

Chr, chromosome; BP, base pair; OR, odds ratio; CI, confidence interval, RAF; risk allele frequency, *N*; the number of participants, Q; Cochran's Q statistic.

Supplementary Table S3. GWAS results of rs762855 in male DGC risk in each cohort and meta-analysis.

rsID	Chr:BP	Risk / Protective alleles	SNUBH				GC_HC				Metaanalysis	
			OR (95% CI)	RAF		*P*-value	OR (95% CI)	RAF		*P*-value	OR (95% CI)	*P*-value
				Case	Control			Case	Control			
rs762855	4:3074795	A / G	1.119 (0.756-1.655)	0.425	0.415	0.575	1.013 (0.831-1.236)	0.406	0.404	0.895	1.034 (0.866-1.235)	0.713

Chr, chromosome; BP, base pair; OR, odds ratio; CI, confidence interval, RAF; risk allele frequency.

Supplementary Table S4. Results of significant variants other than rs762855 in each cohort and the meta-analysis of all three cohorts (SNUBH, GC_HC and SNUBH2_AA).

rsID	Chr:BP	Risk / Protective alleles	R^2	SNUBH		GC_HC		SNUBH2_AA		Metaanalysis (all cohorts)	
				OR (95% CI)	*P*-value	OR (95% CI)	*P*-value	OR (95% CI)	*P*-value	OR (95% CI)	*P*-value
rs3135064	4:3285389	C / T	0.306	1.867 (1.263-2.760)	1.74×10^{-3}	1.611 (1.306-1.986)	8.33×10^{-6}	2.934 (0.846-10.17)	0.0897	1.684 (1.402-2.023)	2.47×10^{-8}
rs28820097	4:3097495	A / G	0.907	2.569 (1.729-3.817)	3.00×10^{-6}	1.479 (1.175-1.862)	8.64×10^{-3}	3.346 (1.032-10.85)	4.42×10^{-2}	1.731 (1.422-2.108)	4.63×10^{-8}

Chr, chromosome; BP, base pair; R^2, correlation with rs762855; OR, odds ratio; CI, confidence interval, RAF; risk allele frequency.

Supplementary Table S5. Independent variants from GWA studies across cancer types and sexes with *P*-values less than 1×10^{-05} and R^2 less than 0.05.

Cancer Type	Sex	rsID	Chr	BP	Risk / Protective Allele	OR	U95	L95	*P*-value	Nearest Gene
DGC	Female	rs762855	4	3074795	A / G	1.758	1.438	2.15	3.91×10^{-08}	*HTT-AS*
DGC	Female	rs78095154	14	53477158	A / C	1.739	1.378	2.195	3.22×10^{-06}	*RP11-368P15.1*
DGC	Female	rs9917107	19	51614461	G / T	2.356	1.641	3.383	3.46×10^{-06}	*CTU1*
DGC	Female	rs866921	6	154611447	A / G	1.609	1.31	1.975	5.66×10^{-06}	*IPCEF1*
DGC	Female	rs17676443	8	22601427	T / G	1.657	1.331	2.061	5.96×10^{-06}	*RP11-459E5.1*
DGC	Female	rs17676443	8	22601427	T / G	1.657	1.331	2.061	5.96×10^{-06}	*PEBP4*
DGC	Female	rs17253263	14	53911906	T / C	1.715	1.353	2.174	8.45×10^{-06}	*AL163953.3*
DGC	Female	rs12189364	5	82932634	T / C	1.587	1.294	1.947	9.27×10^{-06}	*HAPLN1*
DGC	Female	rs56286915	19	44982820	A / G	1.763	1.371	2.267	9.80×10^{-06}	*ZNF180*
DGC	Male	rs11641116	16	83383239	T / C	1.495	1.255	1.779	6.32×10^{-06}	*CDH13*
DGC	Male	rs7247290	19	3731028	G / C	1.545	1.275	1.872	9.07×10^{-06}	*TJP3*
IGC	Female	rs78911886	17	56456802	C / A	3.755	2.292	6.152	1.51×10^{-07}	*BZRAP1-AS1*
IGC	Female	rs78911886	17	56456802	C / A	3.755	2.292	6.152	1.51×10^{-07}	*RNF43*
IGC	Female	rs7674348	4	61482623	T / G	3.995	2.326	6.862	5.21×10^{-07}	*AC095061.1*
IGC	Female	rs11742876	5	12043556	T / C	2.466	1.699	3.578	2.04×10^{-06}	*CTNND2*
IGC	Female	rs34416944	8	12703453	G / A	2.082	1.536	2.822	2.25×10^{-06}	*LINC00681*
IGC	Female	rs4823560	22	46922134	C / A	1.845	1.431	2.378	2.31×10^{-06}	*CELSR1*
IGC	Female	rs12506485	4	190149228	G / A	2.462	1.688	3.591	2.85×10^{-06}	*RP11-706F1.1*
IGC	Female	rs12052135	19	48498337	G/A	1.957	1.467	2.609	4.91×10^{-06}	*ELSPBP1*
IGC	Female	rs7990425	13	77980444	A/G	1.803	1.395	2.331	6.74×10^{-06}	*MYCBP2*

IGC	Female	rs1204375	7	105412253	T/C	1.689	1.34	2.129	9.03×10^{-06}	*ATXN7L1*
IGC	Male	rs28706256	15	80089216	C/A	1.534	1.303	1.807	2.97×10^{-07}	*RNU6-667P*
IGC	Male	rs36178748	15	66394721	G/A	1.49	1.269	1.75	1.14×10^{-06}	*MEGF11*
IGC	Male	rs9967429	18	6008207	C/A	1.626	1.334	1.982	1.53×10^{-06}	*L3MBTL4*
IGC	Male	rs77951789	6	146282338	A/C	1.802	1.41	2.302	2.48×10^{-06}	*SHPRH*
IGC	Male	rs10816699	9	111448306	C/T	1.683	1.355	2.091	2.50×10^{-06}	*RP11-339N8.1*
IGC	Male	rs9459136	6	164974975	A/G	1.44	1.237	1.677	2.69×10^{-06}	*RP11-347L18.1*
IGC	Male	rs9879897	3	187844390	A/C	1.475	1.249	1.742	4.76×10^{-06}	*RP11-430L16.1*
IGC	Male	rs10497881	2	205992964	C/T	1.412	1.215	1.64	6.42×10^{-06}	*PARD3B*
IGC	Male	rs4846246	1	217990625	A/G	0.659	0.55	0.79	6.51×10^{-06}	*SPATA17*
IGC	Male	rs1346562	16	77428247	C/T	1.42	1.218	1.656	7.65×10^{-06}	*ADAMTS18*
IGC	Male	rs140660179	3	140927212	G/C	1.681	1.337	2.114	8.95×10^{-06}	*ACPL2*
IGC	Male	rs78036068	2	76723986	T/A	1.699	1.343	2.148	9.70×10^{-06}	*RN7SKP203*

DGC, diffuse type gastric cancer; IGC, intestinal type gastric cancer; base pair; OR, odds ratio; U95, upper bound of the 95% confidence interval; L95, lower bound of the 95% confidence interval.

Supplementary Table S6. Traits significantly associated with rs762855 according to OpenGWAS.

ID	Trait	*P*-value	β^*
eqtl-a-ENSG00000251075	ENSG00000251075	1.84×10^{-106}	0.2569
eqtl-a-ENSG00000159788	ENSG00000159788	1.59×10^{-84}	-0.2291
ebi-a-GCST90012110	Sex hormone-binding globulin levels adjusted for BMI	5.60×10^{-26}	0.0087
ebi-a-GCST90014004	Gamma glutamyl transferase levels (UKB data field 30730)	5.78×10^{-17}	0.0164
ukb-d-30140_irnt	Neutrophil count	2.31×10^{-15}	0.019
ebi-a-GCST90012108	Sex hormone-binding globulin levels adjusted for BMI	1.30×10^{-14}	0.0081
ukb-d-30000_irnt	White blood cell (leukocyte) count	1.38×10^{-14}	0.0185
ebi-a-GCST90012111	Sex hormone-binding globulin levels	1.90×10^{-14}	0.0072
ebi-a-GCST90012106	Sex hormone-binding globulin levels adjusted for BMI	3.30×10^{-14}	0.0093
ebi-a-GCST90013984	Neutrophil count (UKB data field 30140)	5.67×10^{-14}	0.0162
ebi-a-GCST90018968	Neutrophil count	8.05×10^{-14}	0.0159
ebi-a-GCST90002351	Neutrophil count	1.08×10^{-12}	0.0138
ieu-b-34	neutrophil cell count	1.08×10^{-12}	0.0138
ebi-a-GCST90013976	White blood cell leukocyte count (UKB data field 30000)	1.28×10^{-12}	0.0153
ebi-a-GCST90029003	White blood cell count	1.60×10^{-12}	0.0136
ebi-a-GCST90002374	White blood cell count	9.73×10^{-12}	0.0127
ieu-b-30	white blood cell count	9.73×10^{-12}	0.0127
ebi-a-GCST90018969	Platelet count	1.65×10^{-11}	0.0128
ebi-a-GCST90014014	Triglyceride levels (UKB data field 30870)	1.85×10^{-11}	0.0139
ebi-a-GCST90014011	Sex hormone binding globulin levels (UKB data field 30830)	2.37×10^{-11}	0.0133
ebi-a-GCST90018978	White blood cell count	3.21×10^{-11}	0.013
ukb-d-30870_irnt	Triglycerides	5.15×10^{-11}	0.0154
ukb-d-30080_irnt	Platelet count	7.71×10^{-11}	0.0151
ebi-a-GCST90002395	Mean platelet volume	1.50×10^{-10}	-0.0133
ebi-a-GCST90002346	Mean platelet volume	1.53×10^{-10}	-0.0124
ukb-d-30730_irnt	Gamma glutamyl transferase	1.61×10^{-10}	0.0143
ebi-a-GCST90028999	Platelet count	1.70×10^{-10}	0.0114
ebi-a-GCST90002398	Neutrophil count	2.10×10^{-10}	0.0137
eqtl-a-ENSG00000249673	ENSG00000249673	2.94×10^{-10}	0.075
ieu-b-111	triglycerides	3.00×10^{-10}	0.0126
ebi-a-GCST90012109	Sex hormone-binding globulin levels	3.10×10^{-10}	0.0072
ebi-a-GCST90002412	Low density lipoprotein cholesterol levels	2.00×10^{-09}	0.0116
ebi-a-GCST90093015	Triglycerides to total lipids ratio in very large HDL	2.30×10^{-09}	0.0234
ebi-a-GCST90013996	Aspartate aminotransferase levels (UKB data field 30650)	2.52×10^{-09}	0.0124
ebi-a-GCST90018954	Gamma glutamyl transpeptidase	3.30×10^{-09}	0.0111
met-d-XL_HDL_TG_pct	Triglycerides to total lipids ratio in very large HDL	3.30×10^{-09}	0.0235

ebi-a-GCST90013980	Platelet count (UKB data field 30080)	4.35×10^{-09}	0.0117
ebi-a-GCST90013992	Alanine aminotransferase levels (UKB data field 30620)	6.25×10^{-09}	0.012
met-d-VLDL_size	Average diameter for VLDL particles	6.70×10^{-09}	0.0224
ebi-a-GCST90026654	Estimated glomerular filtration rate (creatinine)	7.93×10^{-09}	0.0013
ebi-a-GCST90093002	Average diameter for VLDL particles	8.70×10^{-09}	0.0224
ebi-a-GCST90093004	Cholesterol levels in very large HDL	9.40×10^{-09}	-0.0216
ebi-a-GCST90002407	White blood cell count	1.00×10^{-08}	0.0123
ebi-a-GCST90018975	Triglycerides	1.07×10^{-08}	0.0115
ebi-a-GCST90093006	Cholesteryl ester levels in very large HDL	1.40×10^{-08}	-0.0212
ukb-d-30870_raw	Triglycerides	1.60×10^{-08}	0.0136
ebi-a-GCST90093008	Free cholesterol levels in very large HDL	1.60×10^{-08}	-0.022
ebi-a-GCST90028996	Mean platelet volume	1.80×10^{-08}	-0.0095
ebi-a-GCST90093010	Total lipid levels in very large HDL	2.20×10^{-08}	-0.021
ukb-d-30830_irnt	SHBG	2.26×10^{-08}	0.0128
met-d-XL_HDL_C	Cholesterol in very large HDL	2.30×10^{-08}	-0.0213
ukb-d-30620_irnt	Alanine aminotransferase	2.82×10^{-08}	0.0125
ebi-a-GCST90093012	Phospholipid levels in very large HDL	2.90×10^{-08}	-0.0208
met-d-XL_HDL_CE	Cholesteryl esters in very large HDL	3.40×10^{-08}	-0.021
met-d-XL_HDL_FC	Free cholesterol in very large HDL	4.20×10^{-08}	-0.0215
ukb-d-30730_raw	Gamma glutamyl transferase	4.33×10^{-08}	0.5523
ieu-a-1239	Years of schooling	4.36×10^{-08}	-0.0093
ukb-b-18377	Leg fat percentage (left)	4.40×10^{-08}	0.0069
met-d-TG_by_PG	Ratio of triglycerides to phosphoglycerides	4.70×10^{-08}	0.0203

[*] β-values were estimated based on risk allele of rs762855 (A).

Supplementary Table S7. Results of Mendelian randomization study based on MR-PRESSO between protein measurements and DGC risk across sex and age.

		NAP		TP		A/G	
		β	*P*-value	β	*P*-value	β	*P*-value
Female	All	0.722	1.04×10^{-2}	1.066	5.23×10^{-3}	-0.659	3.21×10^{-2}
	Age < 50	1.209	1.42×10^{-3}	1.779	2.91×10^{-3}	-0.999	2.06×10^{-2}
	Age ≥ 50	0.501	0.197	0.418	0.408	-0.749	0.106
Male	All	0.019	0.941	0.164	0.630	-0.035	0.906

NAP, non-albumin protein; TP, total protein; A/G, albumin to globulin ratio; β, estimates of causal effects.

Supplementary Table S8. Results of multivariable Mendelian randomization (MR) and MR mediation analyses examining the mediation effects of protein measurement traits between *MSANTD1* expression in stomach tissue and DGC risk in females.

			NAP		TP		A/G	
			β	*P*-value	β	*P*-value	β	*P*-value
All	MVMR	*MSANTD1*	3.944	1.09×10^{-4}	3.605	4.05×10^{-4}	3.908	9.90×10^{-5}
		Trait	0.745	8.02×10^{-3}	1.107	3.69×10^{-3}	-0.645	3.61×10^{-2}
	MR mediation		0.002	0.804	0.053	0.371	-0.007	0.720
Age < 50	MVMR	*MSANTD1*	4.480	2.72×10^{-4}	4.369	2.06×10^{-3}	4.393	5.53×10^{-4}
		Trait	1.209	1.48×10^{-3}	1.800	2.40×10^{-3}	-0.961	2.73×10^{-2}
	MR mediation		0.000	0.987	0.023	0.686	-0.046	0.464
Age ≥ 50	MVMR	*MSANTD1*	5.683	5.43×10^{-6}	5.756	2.60×10^{-6}	5.704	2.21×10^{-5}
		Trait	0.546	0.151	0.425	0.381	-0.766	0.091
	MR mediation		0.001	0.844	0.040	0.571	-0.003	0.881

MVMR, multivariable Mendelian randomization; NAP, non-albumin protein; TP, total protein; A/G, albumin to globulin ratio; β, estimates of causal effects.

Supplementary Table S9. Significant variants of GWAS for DGC risk in females less than 50 years old in each cohort and meta-analysis of the two cohorts.

**rsID**	**Chr:BP**	**Risk / Protective alleles**	**SNUBH**				**GC_HC**				**Metaanalysis**	
			OR (95% CI)	RAF Case	RAF Control	*P*-value	OR (95% CI)	RAF Case	RAF Control	*P*-value	OR (95% CI)	*P*-value
rs11648430	16:29022557	G / A	3.158 (1.563-6.382)	0.531	0.322	1.35×10^{-3}	2.938 (1.837-4.699)	0.331	0.162	6.86×10^{-6}	3.003 (2.029-4.445)	3.81×10^{-8}
rs12923895	16:29022380	T / C	3.158 (1.563-6.382)	0.531	0.322	1.35×10^{-3}	2.914 (1.824-4.656)	0.331	0.163	7.75×10^{-6}	2.986 (2.019-4.416)	4.31×10^{-8}

Chr, chromosome; BP, base pair; OR, odds ratio; CI, confidence interval, RAF; risk allele frequency.

Supplementary Table S10. Traits significantly associated with rs11648430 according to OpenGWAS. βs were estimated based on risk allele of rs11648430 (G).

**ID**	**Trait**	***P*-value**	β
eqtl-a-ENSG00000178952	ENSG00000178952	1.29×10^{-263}	0.4131
eqtl-a-ENSG00000169682	ENSG00000169682	4.68×10^{-191}	0.3551
eqtl-a-ENSG00000176476	ENSG00000176476	3.59×10^{-23}	-0.1226
eqtl-a-ENSG00000205609	ENSG00000205609	1.08×10^{-17}	0.106
eqtl-a-ENSG00000197165	ENSG00000197165	4.55×10^{-17}	0.1039
ebi-a-GCST90002334	Mean corpuscular volume	1.22×10^{-13}	0.0145
eqtl-a-ENSG00000184110	ENSG00000184110	9.14×10^{-12}	0.0845
ebi-a-GCST90002392	Mean corpuscular volume	2.00×10^{-11}	0.0148
eqtl-a-ENSG00000188322	ENSG00000188322	2.38×10^{-10}	-0.0785
ebi-a-GCST90025963	Mean corpuscular volume	7.20×10^{-10}	0.0115
ebi-a-GCST90025975	Mean reticulocyte volume	5.10×10^{-09}	0.0117
eqtl-a-ENSG00000196502	ENSG00000196502	8.78×10^{-09}	0.0713
ebi-a-GCST90002363	Red blood cell count	3.92×10^{-08}	-0.011
